# Arapan-S: a fast and highly accurate whole-genome assembly software for viruses and small genomes

**DOI:** 10.1186/1756-0500-5-243

**Published:** 2012-05-16

**Authors:** Mohammed Sahli, Tetsuo Shibuya

**Affiliations:** 1 Department of Computer Science, Graduate School of Information Science and Technology, University of Tokyo, 7-3-1 Hongo, Bunkyo-ku, Tokyo, 113-0033, Japan; 2 Human Genome Center, Institute of Medical Science, University of Tokyo, 4-6-1 Shirokanedai, Minato-ku, Tokyo, 108-8639, Japan

## Abstract

**Background:**

Genome assembly is considered to be a challenging problem in computational biology, and has been studied extensively by many researchers. It is extremely difficult to build a general assembler that is able to reconstruct the original sequence instead of many contigs. However, we believe that creating specific assemblers, for solving specific cases, will be much more fruitful than creating general assemblers.

**Findings:**

In this paper, we present Arapan-S, a whole-genome assembly program dedicated to handling small genomes. It provides only one contig (along with the reverse complement of this contig) in many cases. Although genomes consist of a number of segments, the implemented algorithm can detect all the segments, as we demonstrate for *Influenza Virus A*. The Arapan-S program is based on the de Bruijn graph. We have implemented a very sophisticated and fast method to reconstruct the original sequence and neglect erroneous *k*-mers. The method explores the graph by using neither the shortest nor the longest path, but rather a specific and reliable path based on the coverage level or *k*-mers’ lengths. Arapan-S uses short reads, and it was tested on raw data downloaded from the NCBI Trace Archive.

**Conclusions:**

Our findings show that the accuracy of the assembly was very high; the result was checked against the European Bioinformatics Institute (EBI) database using the NCBI BLAST Sequence Similarity Search. The identity and the genome coverage was more than 99%. We also compared the efficiency of Arapan-S with other well-known assemblers. In dealing with small genomes, the accuracy of Arapan-S is significantly higher than the accuracy of other assemblers. The assembly process is very fast and requires only a few seconds.

Arapan-S is available for free to the public. The binary files for Arapan-S are available through http://sourceforge.net/projects/dnascissor/files/.

## Background

Sequencing technologies have been providing us with thousands of sets of genomic reads (sometimes called fragments or segments), with each set being taken from a specific genome. Bringing these reads all together in order to reconstruct the original sequence (the genome) is commonly known as the (whole-) genome assembly problem. This problem has been studied extensively and many assemblers, along with some assembly models, have been proposed. Most models are based either on the overlap graph approach or the de Bruijn graph-based approach. The overlap graph is a graph whose nodes represent the genomic reads, while its edges correspond to the overlaps of these reads. It was the pillar of the first assemblers that appeared on the market, such as: TIGR [1], CAP3 [2], PCAP [3], the string graph of Myers [4] and MIRA [5]. The second category of assemblers is based on the de Bruijn graph, in which the nodes represent the substrings (*k*-mers) of the genomic reads (which are of the same length), while the edges correspond to the overlaps of these substrings. The de Bruijn graph has become the standard pillar of the so-called “de novo” assemblers. Some of the assemblers based on this approach include: Euler assembler [6], SSAKE [7], EULER-SR [8], Velvet [9,10], ALLPATHS [11,12], ABySS [13], and SOAPdenovo [14]. Although the assemblers share the same graph structure, they use different (but sometimes similar) algorithms to walk through the graph. To our knowledge, there is no proof that the shortest or the longest path, or the Hamiltonian or Eulerian paths will represent the genome in its natural form; therefore, we developed an algorithm that selects only the reliable nodes in the de Bruijn graph in order to reconstruct the original sequence of small genomes or long contigs when the graph is sparse.

Because of the diversity of genomes, creating a general assembler that is able to solve all cases will not be as effective and fast as a specific assembler that focuses on solving particular cases. For instance, ploidy can be a serious problem when dealing with plant genomes in which tetraploidy is common. Concerning very small genomes, we believe that we can improve the accuracy of assembly of such genomes by creating an assembler that is devoted to solving small genomes. That is the reason we aimed to create an assembler (named Arapan-S) dedicated to solving small genomes. As a result, the Arapan-S assembler was able to reconstruct one very highly accurate supercontig in most cases. To check the accuracy of Arapan-S, we performed a BLAST sequence similarity search against the EBI (European Bioinformatics Institute) database, which includes the complete genomes of our dataset. This analysis showed that the Arapan-S assemblies were more than 99% accurate. We also compared Arapan-S with other well known assemblers in the assembly of viral genomes.

## Findings

### Arapan-S parameters

Arapan-S was written in C/C++ language under a programming framework called Qt on a 64-bit Linux machine and was also compiled in Windows. The input data must represent each *k*-mer (i.e. de Bruijn sequence), along with its frequency in the same line, separated by a whitespace character. Note that all frequency values of generated *k*-mers are based on the coverage level of the dataset. In other words, we have used such frequency values instead of the coverage value. A tool called *kmerBuilder*, which is one of several assembly pipelines included in the Arapan software package, can generate *k*-mer files for Arapan-S (i.e. the dataset must be prepared independently from our assembler). The project acronym (Arapan) represents our primary goal to produce a software system that includes a set of open-source tools dedicated to solving and analyzing the whole genome assembly problem.

The Arapan-S assembler is very sensitive to the length of *k* of short reads, and because of its architecture our tool always tries to find one supercontig along with its reverse complement. Nevertheless, if the length of *k* is very short, Arapan-S will encounter some difficulties in constructing the original sequence. Also, if *k* is very long, the result of the assembly will not be significant. There is always a trade-off between the specificity and sensitivity of choosing the length of *k*. By experiment, the most appropriate value of *k* is when 20 ≤ *k* ≤ 35.

Arapan-S has only one parameter, which is the merging function: the frequency function or the *k*-mer length function. The graphical user interface of Arapan-S represents this parameter by a check-box. During the experiments, it was preferable to choose the frequency function, since it usually leads to a more accurate result. We have considered the frequency function to be the only objective function in our experiments.

### BLAST similarity search

We downloaded some real datasets from the NCBI Trace Archive (ftp://ftp.ncbi.nih.gov/pub/TraceDB/). The data were cleaned and prepared by a trimming tool (http://sourceforge.net/projects/dnascissor/files/DNA%20Scissor/). A minimum quality value cut-off of 20 (i.e. the accuracy of the base call was 99%) was set for most of the genomes, and the low-quality end regions were trimmed at the 5′-end and 3′-end of every read. The short reads (*k*-mers) were generated by the same trimming tool for each set of reads. The Arapan-S assembler was very fast, used less memory and provided us with one supercontig along with its reverse complement in many cases. For checking the accuracy of our assembler, we searched for the obtained supercontigs (the complete genome) on the EBI database using the NCBI BLAST Similarity Search. The input data are given in Table 1, while Table 2, Table 3, Table 4 and Table 5 show the results.

**Table 1:** The input data include seven Virus Genomes

**Species**	**Accession number**	**Number of reads**	**Read average length (bp)**
Bovine Respiratory Coronavirus AH187	FJ938065.1	635	995
Calf-giraffe Coronavirus US/OH3/2006	EF424624.1	548	935
Waterbuck Coronavirus US/OH-WD358-TC/1994	FJ425184.1	576	984
White-tailed Deer Coronavirus US/OH-WD470/1994	FJ425187.1	503	918
Antelope coronavirus US/OH1/2003	EF424621.1	616	991
Influenza A Virus (A/Memphis/1/71(H3N2))	From CY006211.1 To CY006218.1	132	570
Influenza A Virus (A/Swine/Colorado/1/77/(H3N2))	Q288Y7 (EBI)	159	596
Influenza A Virus (A/Weiss/43/(H1N1))	From CY009452.1 To CY009459.1	168	519

We considered eight viruses. The genome of each Influenza A Virus consists of eight segments while the others have only one long segment. The datasets represent Sanger reads. The raw data were downloaded from NCBI Trace Archive (ftp://ftp.ncbi.nih.gov/pub/TraceDB/.)

**Table 2:** The Alignment Results By Using the EBI database (BLAST Similarity Search) on seven Virus Genomes

**Species**	**Total length** **(bp)**	**Genome length** **(EBI)**	**Alignment score**	**Identities**	**Expect value** ***E*()**
Bovine Respiratory Coronavirus AH187	30936	30969	30875	99.803%	0.0
Calf-giraffe Coronavirus US/OH3/2006	30831	30979	30762	99.776%	0.0
Waterbuck Coronavirus US/OH-WD358-TC/1994	30995	30995	30934	99.803%	0.0
White-tailed Deer Coronavirus US/OH-WD470/1994	31018	31020	30957	99.803%	0.0
Influenza A virus (A/Memphis/1/71(H3N2))	12598	13397	12503	99.246%	0.0
Influenza A Virus (A/Swine/colorado/1/77/(H3N2))	13019	13304	12969	99.616%	0.0
Influenza A Virus (A/Weiss/43/(H1N1))	13300	13371	13208	99.308%	0.0

The Total length is the length of the obtained result, while Genome length (EBI) is the genome’s supposed length according to the EBI database. The values of Identities were calculated by dividing Alignment scores by the corresponding Total lengths. The Expect-value was calculated by EBI’s NCBI BLAST Similarity Search engine.

**Table 3:** Comparison of Arapan-S with ABySS, SSAKE, Velvet, QSRA, Minimus and Mira assemblers on four Benchmark Virus Genomes

**Species**	**Assembler**	**Contigs ≥ 800 bp**	**Total** **length**	**Mean size (bp)**	**N50 (bp)**	**Largest contig (bp)**	**Genome coverage (%)**
**Bovine Respiratory Coronavirus AH187**	**Arapan-S**	1	30937	30937	30937	30937	99.90
	**ABySS**	1	30924	30924.00	30924	30924	99.85
	**SSAKE**	9	27428	3047.56	3447	9868	88.57
	**Velvet**	3	30951	10317.00	25461	25461	99.94
	**QSRA**	8	29617	3702.125	-	11695	95.63
	**Minimus**	1	31026	31026	31026	31026	100.18
	**Mira**	8	28803	3600.37	3192	12305	93.51
**Calf-giraffe Coronavirus** **US/OH3/2006**	**Arapan-S**	1	30836	30836	30836	30836	99.53
	**ABySS**	2	30652	15326.00	18956	18956	98.94
	**SSAKE**	11	17005	1545.91	892	2683	54.89
	**Velvet**	3	30951	10317.00	25461	25461	99.91
	**QSRA**	2	2107	1053.5	-	1173	6.80
	**Minimus**	1	30979	30979	30979	30979	100.00
	**Mira**	5	33850	6770	20763	20763	109.28
**Waterbuck Coronavirus US/OH-WD358-TC/1994**	**Arapan-S**	1	30995	30995.00	30995	30995	100.00
	**ABySS**	1	30944	30944.00	30944	30944	99.86
	**SSAKE**	13	21780	1675.38	1063	5343	70.27
	**Velvet**	8	12505	1563.12	967	2162	40.34
	**QSRA**	5	4638	927.6	-	1174	14.96
	**Minimus**	1	30995	30995	30995	30995	100.00
	**Mira**	6	34011	5668.5	10510	10983	109.73
**White-tailed Deer Coronavirus US/OH-WD470/1994**	**Arapan-S**	1	31018	31018.00	31018	31018	99.99
	**ABySS**	2	30943	15471.50	21535	21535	99.75
	**SSAKE**	5	13925	2785.00	956	6100	44.89
	**Velvet**	10	17800	1780.00	1090	3430	57.38
	**QSRA**	8	7422	927.75	-	1323	23.93
	**Minimus**	1	31019	31019	31019	31019	100.00
	**Mira**	10	34892	3489.2	6174	9191	112.48

Only contigs whose lengths ≥ 800 were selected. When the assembler generated only one contig, the N50 value and the mean size are equal to the size of the corresponding contig. Genome coverage was calculated by dividing the total length by the genome length (EBI).

**Table 4:** Comparison of Arapan-S with all the assemblers on Three Genomes Composed of eight Segments

**Species**	**Assembler**	**Contigs ≥ 400 bp**	**Total Length**	**Mean size (bp)**	**N50 (bp)**	**Largest contig (bp)**	**Genome coverage (%)**
**Influenza A Virus** **A/Memphis/1/71(H3N2)**	**Arapan-S**	8	12598	1574.75	1584	2311	94.03
	**ABySS**	14	12897	921.21	1280	1801	96.27
	**SSAKE**	1	555	555.00	-	555	4.14
	**Velvet**	14	10774	769.57	789	1781	80.42
	**QSRA**	17	12570	739.41	700	1828	93.83
	**Minimus**	9	13156	1461.78		2242	98.20
	**Mira**	14	14399	1028.50	1396	2080	107.48
**Influenza A Virus** **A/Swine/Colorado/1/77/(H3N2)**	**Arapan-S**	8	13120	1640	2151	2310	99.13
	**ABySS**	9	12478	1386.44	1634	2262	93.79
	**SSAKE**	6	4287	714.50	-	1409	32.22
	**Velvet**	12	9783	815.25	494	1867	73.53
	**QSRA**	16	9400	587.50	468	1200	70.65
	**Minimus**	8	13325	1665.62	2199	2309	100.16
	**Mira**	10	14678	1467.80	1780	2371	110.33
**Influenza A Virus** **A/Weiss/43/(H1N1)**	**Arapan-S**	8	13300	1662.50	2194	2313	99.47
	**ABySS**	11	13108	1191.64	1716	2274	98.03
	**SSAKE**	3	1616	538.67	-	572	12.09
	**Velvet**	9	9764	1084.89	1006	1696	73.02
	**QSRA**	16	11755	734.69	573	1916	87.91
	**Minimus**	8	13369	1671.12	2194	2313	99.98
	**Mira**	11	15139	1376.27	1583	2359	113.22

Only contigs whose lengths ≥ 400 were selected. Each species has eight segments that constitute its genome.

**Table 5:** **Comparison of Arapan-S with all QSRA, Minimus and Mira assemblers on*Antelope coronavirus US/OH1/2003*genome**

**Species**	**Assembler**	**Contigs ≥ 400 bp**	**Total Length**	**Mean size (bp)**	**N50 (bp)**	**Largest contig (bp)**	**Genome coverage**
**Antelope coronavirus US/OH1/2003**	**Arapan-S**	1	26280	26280	26280	26280	98.89
	**QSRA**	0	0	0	0	0	0
	**Minimus**	1	30994	30994	30994	30994	116.63
	**Mira**	5	33793	6758.6	31042	31042	116.81

Only contigs whose lengths ≥ 400 were selected.

The total length of each genome was very close to the genome length obtained from the EBI database, and yielded very high identities (Table 2). Moreover, to show the robustness of Arapan-S, we compared its results to other well-known assemblers: ABySS-1.2.7 [13], SSAKE 3.7 [7], Velvet 1.1.3 [9,10] and QSRA [15]. The Overlap-Layout-Consensus-based assemblers that were included for comparison were: Minimus [16] and Mira [5,17]. The selected version of each assembler was the latest release, except for the SSAKE assembler for which we chose the release SSAKE 3.7 instead of SSAKE 3.8 because of installation problems. All assemblers have been run with default parameters.

### Comparison

Because of its architecture (de Bruijn graph), Arapan-S is classified as a de novo assembler. However, since our datasets are Sanger reads, we compared our assembler with de novo assemblers and also Overlap-Layout-Consensus assemblers. Note that the current version of QSRA assembler is not able to deal with different read lengths. To solve this problem we used our tool, *kmerBuilder,* which is also in the Arapan package, to generate reads of the same length (200 bp for QSRA) from shotgun data.

#### De novo assembler competitors

Concerning the de novo assemblers, the most competitive assembler to Arapan-S was ABySS in Table 3. As with Arapan-S, ABySS was also able to produce only one supercontig for the *Bovine Respiratory Coronavirus AH187* genome and the *Waterbuck Coronavirus US/OH WD358 TC/1994* genome. However, in contrast to ABySS, Arapan-S achieved the greatest genome coverage and only one supercontig in all cases. Since Arapan-S generated only one contig in all cases, it produced the largest contigs compared to other assemblers. In contrast, the other assemblers generated more contigs and SSAKE had the lowest genome coverage every time and more contigs most of the time. QSRA also did not work well with small genomes.

The *Influenza A Virus* genome consists of eight segments (http://bioafrica.mrc.ac.za/rnavirusdb/virus.php?id=335341). Table 4 shows that Arapan-S was able to detect the eight contigs of different genomes of type Influenza A Virus. According to our empirical results, SSAKE failed to deal with small viral genomes. N50 values of SSAKE were not computed because its results did not cover half of the entire genome. ABySS was again the second best assembler after Arapan-S. However, our assembler succeeded in determining the eight segments of each genome, such that its N50 values, as well as the largest contig, were always the highest compared to other assemblers.

#### Overlap-layout-consensus competitors

Among the Overlap-Layout-Consensus-based assemblers, Arapan-S was comparable to Minimus. Minimus failed in one case, *Influenza A Virus A/Memphis/1/71(H3N2)*, in which it produced nine contigs instead of eight (Table 4). Our assembler showed good approximation compared to Minimus for the *Antelope coronavirus US/OH1/2003* genome (Table 5). They achieved almost the same result for the *Waterbuck Coronavirus US/OH-WD358-TC/1994* and the *White-tailed Deer Coronavirus US/OH-WD470/1994* genomes. On the other hand, Mira did not work well with small genomes, as shown in Tables 3, 4 and 5.

## Discussion

We have relied on only one objective function “*the frequency function”* for the sequence assembly algorithm. In fact, one may also consider another function, which is, “the k-mer length function”, $g(L)=\Sigma_{i}{}^{N}{=}_{1}a_{i}l_{i}\text{,}$ such that $L=\left\{l_{1},l_{2},K,l_{N}\right\}$ such that is the set of k-mer lengths. This function is based on the assumption that nodes whose *k*-mers have longer, relative to shorter, lengths are more probably generated from trustworthy consecutive nodes, that is to say, a chain that has fewer or no sequencing errors. However, we have considered only the frequency function in the analysis presented here.

In the case of non-uniform coverage of some areas in the genome [18], the frequency function may suffer from less accuracy. On the other hand, we believe that the *k*-mer length function can be a good choice in the case of coverage non-uniformity. Building an algorithm that combines the two objective functions and switches from one to another may lead to more accurate results. Creating such an effective algorithm is an important issue for future research.

Another thing that can be said about the objective function is that the assembly algorithm does not look for the optimal solution. As a matter of fact, the algorithm starts at a determined node whose associated *k*-mer has the longest length, then starts going forward and backward in the graph selecting nodes that have the highest scores (greatest frequency values) locally in order to construct a contiguous path in a given connected component.

We have noticed that most genome assemblers, which were built for tackling medium or large genomes, could not successfully deal with tiny and small genomes. Arapan-S, ABySS and Minimus were able to deal with such cases. In future work a comparison would be worthwhile for all genome assemblers to determine the efficiency field of each set of assemblers.

Since our aim was creating a genome assembler for tackling only tiny genomes, dealing with repeats was not an essential task, since they do not regularly appear in very small genomes and the confrontation with tandem repeats does not generally mislead the assembly process (according to our experience). However, in the future, we aim to build another version of the Arapan-S assembler that can handle longer genomes.

## Conclusions

According to our experiments, we have found that general assemblers are not always as effective as the Arapan-S assembler in dealing with tiny genomes. We have used only long reads in our experiments, because the raw data of small genomes can be easily found in the NCBI Trace Archive. However, our assembler can work with any other sequencing technology, such as Illumina/Solexa, SOLiD and 454 sequencing technologies. The raw data are converted into a set of *k*-mers by kmerBuilder (http://sourceforge.net/projects/dnascissor/files/kmerBuilder/). The user can run Arapan-S assembler by providing it with the *k*-mer file. This feature represents another advantage of our assembler compared to other assemblers. Arapan-S is fast and uses less memory. However, because we are dealing with small genomes, the time and space complexities of all assemblers were negligible. Our assembler is not designed to be applied to medium or large genomes.

## Methods

The assembly process consists of four major phases. In the first phase, the de Bruijn graph is straightforwardly constructed. The second phase (called the *cleaning process*) is a very important step in which the graph is simplified as much as possible by collapsing paths, removing tips and solving bubbles, as well as handling a few other different structures in the graph. In the third phase the graph components are detected before starting the assembly algorithm in the fourth step.

**Figure 1:**
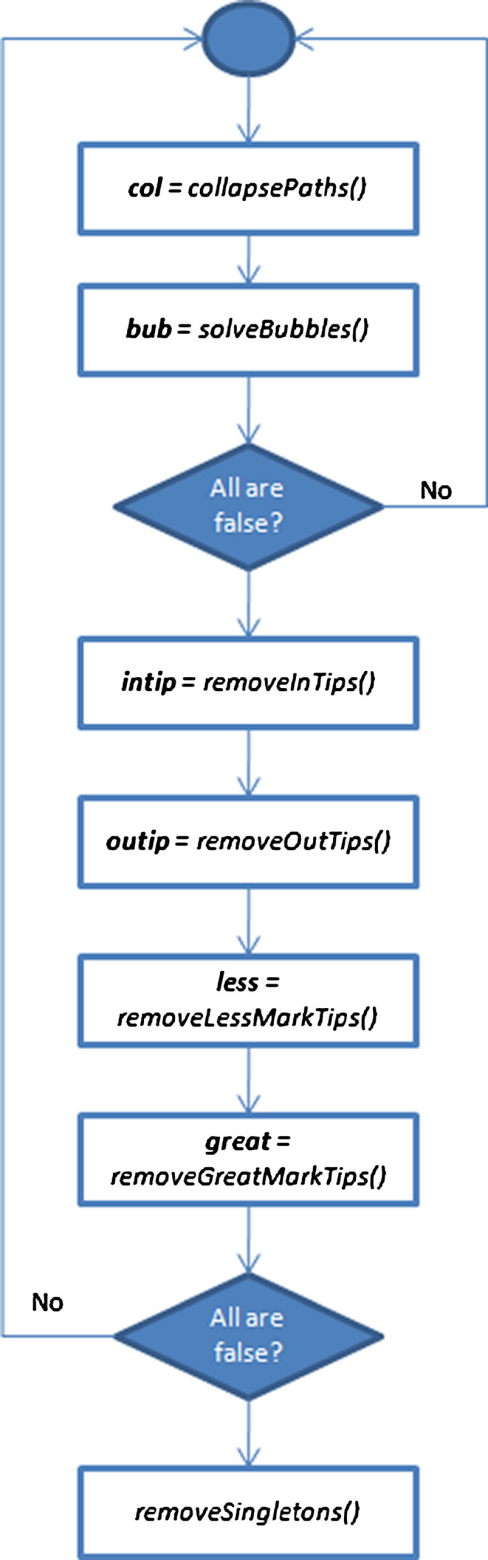
**Flowchart.** The different phases of the cleaning algorithm.

Our algorithm differs from previous works in the following ways:

1. The cleaning process simplifies the graph by a few iterations without incorporating time-consuming algorithms, such as the Dijkstra-like breadth-first search in Velvet [9,10] and the Dijkstra algorithm in SOAPdenovo [14].

2. An algorithm was created to solve only simple bubbles (Figure 1), but by involving other algorithms (i.e. paths collapsing, tips, etc.) all complex bubbles are solved after a few iterations of the cleaning algorithm.

3. The assembly algorithm uses the frequency values and lengths of *k*-mers in order to construct contigs as will be described below.

Most de novo assemblers focus on solving large genomes; this involves implementing time-consuming and very complicated algorithms. As a result, the construction of contigs becomes stricter, though this is not the case for small genomes, as shown in the results section.

### Input data and graph construction

The entire dataset of *k*-mers is recorded using hash tables in order to speed up further operations. The reverse complements are also recorded without binding them with their original *k*-mers. All we need is a linear algorithm for constructing the de Bruijn graph. Since the alphabet is composed of four nucleotide letters, each *k*-mer will be connected to four *k*-mers at most. All *k*-mers that include unknown ‘N’ nucleotides are discarded. The pseudo-code of the algorithm is shown below:

1. **deBruijnGraphBuilder(**HashTable *kmerList***, integer***K***)**

2. **Integer***N* :=|*kmerList*|; //the size of *kmerList*

3. **String***temp*;

4. **for***i*:=1 **to***N***do**

5. **begin**

6. *temp* := *kmerList*[*i*][1..*K*−1];

7. //forward connection

8. **if***temp*+“A” *kmerList***then** createArc( *i*, *kmerList*.IndexOf(*temp*+“A”));

9. **if***temp*+“T” *kmerList***then** createArc( *i*, *kmerList*.IndexOf(*temp*+“T”));

10. **if***temp*+“C” *kmerList***then** createArc( *i*, *kmerList*.IndexOf(*temp*+“C”));

11. **if***temp*+“G” *kmerList***then** createArc( *i*, *kmerList*.IndexOf(*temp*+“G”));

12. //backward connection

13. **if** “A”+ *temp kmerList***then** createArc(*kmerList*.IndexOf(“A”+ *temp*), *i* );

14. **if** “T”+ *temp kmerList***then** createArc(*kmerList*.IndexOf(“T”+ *temp*), *i* );

15. **if** “C”+ *temp kmerList***then** createArc(*kmerList*.IndexOf(“C”+ *temp*), *i* )

16. **if** “G”+ *temp kmerList***then** createArc(*kmerList*.IndexOf(“G”+ *temp*), *i*);

17. **end**

Let *K* be the length of the short reads. The variable *temp* will contain the first prefix of a given *K*-mer whose length is *K* − 1. The algorithm computes the out-neighbours in the forward orientation, and the in-neighbours in the opposite direction.

### Cleaning process (simplifying the graph and solving errors)

**Figure 2:**
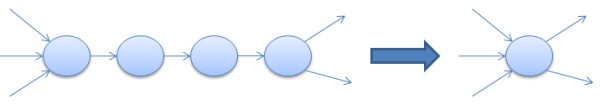
**Switch node.** All contiguous nodes are merged in one node. This operation is named “*The path collapsing.*”

The raw DNA data always suffer from errors, and since the de Bruijn graph is based on the exact matching of *k*-mers, error correction (or removal) becomes very important to the use of such graphs in representing and analyzing sequencing data. The coverage plays a vital role in guiding the cleaning and assembly algorithms to a more accurate result. After constructing the graph, some erroneous *k*-mers appear in the graph in different forms. The most common forms are the so-called “Tips, Bubbles and Chimeric connections”. However, while analyzing the graph, we found other forms as well. We have implemented an iterative algorithm that reduces the graph to its maximum simplification. The pseudo-code of the algorithm is shown below and its flowchart is given in Figure 2.

1. ***cleaningAlgorithm****()*

2. ***Boolean****col, bub, intip, outip, less, great;*

3. ***Begin***

4. ***do***

5. *col := collapsePaths();*

6. *bub := solveBubbles();*

7. ***if****col==false****and****bub==false****then***

8. ***begin***

9. *intip := removeInTips();*

10. *outip := removeOutTips();*

11. *less := removeLessMarkTips();*

12. *great := removeGreatMarkTips();*

13. ***if****intip==false****and****outip==false****and***

14. *less ==false****and****great==false****then****stop;*

15. ***end***

16. ***while****(true)*

17. *removeSingletons();*

18. ***End***

The *collapsePaths*() procedure will return false if it does not collapse any path, otherwise, it returns true. The other procedures behave exactly as *collapsePaths*() does. We will hereafter explain each procedure invoked by the cleaning algorithm.

#### Path collapsing

To simplify and shrink the graph before applying any cleaning procedure, a path collapsing algorithm should be run immediately after constructing the graph.

**Figure 3:**
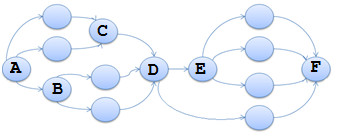
**Bubbles.** This figure illustrates three simple bubbles and two complex bubbles. Simple bubbles are A-C, B-D and E-F. The first complex bubble starts at A and ends at D while the second one starts at D and ends at F. (X-Y is the subgraph that starts at X and ends at Y). Complex bubbles are solved by executing the simple bubble-solving algorithm and path-collapsing algorithm.

A path is a chain of nodes. Two nodes *X* and *Y* are merged if the node *X* has only one outgoing arc connected to the node *Y* that has only one incoming arc. Their corresponding *k*-mers must be concatenated accordingly. Most of the resulting nodes (we call them *switch nodes*) are seen in Figure 3.

#### Bubble solving

In genome assembly, a bubble appears where two sequences initially align, then diverge in the middle, and align again at the end. Bubbles are caused by repeats or heterozygotes of diploid chromosomes [14], or created by errors or biological variants, such as SNPs, diploids or cloning artefacts prior to sequencing.

A path is a chain of nodes in a graph. We call a path a simple path if each internal node (i.e., each node between the start node and the end node of the path) has one outgoing edge and one incoming edge. A bubble is a subgraph that consists of multiple simple paths all of which share the same start node and the same end node. In the original graph, the start node must not have any outgoing edges other than those in the bubble, and the end node must not have any incoming edges other than those in the bubble.

In Velvet [9,10], detection of bubbles was done by an algorithm based on a Dijkstra-like breadth-first search called “The Tour Bus Algorithm”. Similarly, Dijkstra’s algorithm is also used to detect bubbles in SOAPdenovo [14], in which the detected bubbles are merged into a single path if the sequences of the parallel paths are very similar; that is, had fewer than four base pairs difference with more than 90% identity.

In Arapan-S, all bubbles will be relaxed by combining all the cleaning procedures and without incorporating a time-consuming algorithm. After collapsing all paths, bubbles will appear in the graph as shown in Figure 1. The node with a high coverage will not be removed from the bubble (However, the algorithm can also be parameterized to keep only the node that has the maximum *k*-mer’s length instead of high coverage).

#### Tips removal

**Figure 4:**
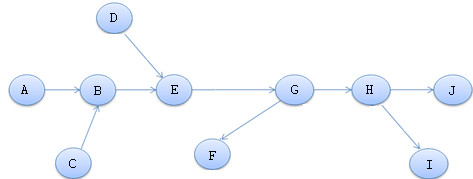
**Tips.** This figure shows some tips (i.e. C, D, F and I).

**Figure 5:**
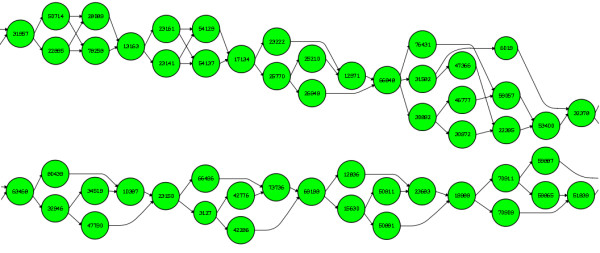
**Graph visualization.** A part of two connected components of the white tailed deer corona virus genome graph after running the cleaning algorithm. Nodes represent *k*-mers and arrows represent the overlaps between *k*-mers. This picture was taken from the aiSee graph visualization software (www.aisee.com.)

Tips generally result from errors at the end of reads. In the graph, a tip is a node connected only on one end (Figure 4). In Velvet, a tip is removed if it is shorter than 2 *k* (*k* is chosen for the *k*-mer). After removing tips, new paths will appear again in the graph. Almost all the remaining nodes’ degrees are ≥ 2. We will hereafter call such nodes: *switch nodes*. The result of the cleaning process will be similar to what is shown in Figure 5.

### Connected components detection

Once the graph is reduced and contains only switch nodes, we start determining the connected components of the graph. There are two cases in which we need to determine the connected component. The first case is the nature of the *k*-mers and their reverse complements. Since each *k*-mer was recorded along with its reverse complement, we will obtain a graph composed of two subgraphs, one being the reverse of the other. The second case is the sparseness of the graph, especially when the initial *k*-mer length is a bit longer. Our assembly algorithm can run on every connected component of the graph. Detection of these components can lead the assembly algorithm to be run in parallel. The breadth-first search or depth-first search can be applied to find the connected components in linear time. The search begins at an arbitrary node *v* from which the entire connected component including *v* will be detected. A loop through all nodes of the graph must be implemented in order to find all the connected components. The loop runs until no visited node can be found. The pseudo-code of the modified algorithm is shown as follows:

1. ***connectedComponent(VertexSet****V****, EdgeSet****E****, Node****a****)***

2. ***Set****X;*

3. ***Boolean****visited[|V|];*

4. *//Step 1*

5. $X:=X\cup \left\{a\right\}\text{;}$

6. $visited\left\lfloor x\right\rfloor :=false,\forall x\in V\text{;}$

7. *//Step 2*

8. ***while***∃x∈X|visitedx=false***do***

9. ***begin***

10. ∃x∈X|visitedx=false;

11. $X:=X\cup \left\{y\right\},\forall \left(x,y\right)\in Eor\left(y,x\right)\in E|y\notin X\text{;}$

12. ***end***

13. ***return****X;*

The idea of this algorithm is to traverse the graph from an arbitrary node *a*, mark it as a visited node and record its neighbors in the set *X*. The same job is done for the recorded nodes until there are no visited nodes in the set *X*. The algorithm returns the connected component engendered from the node *a*. To find all connected components we apply the following algorithm:

1. ***allComponents(VertexSet****V****, EdgeSet****E****)***

2. ***SetList****C;*

3. ***Set****X’ ;*

4. ***Integer****i;*

5. *//Step1*

6. X':=V;

7. i:=1;

8. *//Step 2*

9. ***while***X'≠Ø***do***

10. ***begin***

11. *select an arbitrary x∈X’;*

12. $C_{i}:=connectedComponent(G,x)\text{;}$

13. $X':X'-C_{i}\text{;}$

14. i:=i+1;

15. ***end***

16. ***return****C;*

We only need to select an arbitrary node *x* and determine, due to the *connectedComponent*() procedure, the connected component *C*_*i*_ having *x*. The determined component’s nodes will be removed from the *X’* (Line 14). The same operation is performed until no connected components can be detected.

### Assembly algorithm

Once the connected components are detected, we run the assembly algorithm for each component. The assembly algorithm can be run by using one of two parameters: the coverage (*k*-mer’s frequency), and the *k*-mer lengths. The latter parameter is obtained by the cleaning process, which provides us with *switch nodes* whose corresponding *k*-mers have longer lengths due to the merging process.

Most of the previous work on genome assembly has the following assumption: given a set of reads, the objective of the assembly program is to minimize the length of the assembled genome [18]. However, according to our knowledge, there is no proof that the shortest path can always faithfully represent the genome. The same can be concluded concerning the longest path, the Hamiltonian path and the Eulerian path.

The assembly algorithm is a greedy function. It traverses the graph by selecting only the nodes whose frequency values are higher. We have chosen this strategy by assuming that *k*-mers, which are characterized by high frequency values, are more likely to be free of sequencing errors (we call it “*frequency function*”). All procedures of the assembly algorithm are given as follows:

1. ***stringPath( SetC)***

2. ***Ordered Set****path;*

3. ***Set****P, Visited;*

4. ***Node****u, v;*

5. *//Step1: preprocessing*

6. *u := the index of the node which have the longest k-mer length.*

7. v:=u;

8. $path:=path\cup \left\{u\right\}\text{;}$

9. Visited: =Ø

10. *//Step 2: forward direction*

11. ***do forever***

12. ***begin***

13. *P := out_neighbors(u) − Visited;*

14. Visited:=Visited∪P*;*

15. ***if****P=*Ø ***then****stop;*

16. *u := bestNeighbor(u, P);*

17. $path:=path\cup \left\{u\right\}\text{;}$

18. ***End***

19. *//Step 3: backward direction*

20. ***do forever***

21. ***begin***

22. *P := in_neighbors(v) − Visited;*

23. Visited:=Visited∪P;

24. ***if****P=Ø****then****stop;*

25. *v := bestNeighbor(v, P);*

26. $path:=\left\{v\right\}\cup path\text{;}$

27. ***end***

28. ***return****path;*

The set *C* represents a connected component of the graph. The resulting path is kept in the ordered set *path.* After variables initialization, the algorithm goes in a forward direction selecting the best out-neighbors. In the last step, it goes backwards selecting the best in-neighbors. The *bestNeighbor*() function is the current node and the set of its in- or out-neighbors. Since each node could be connected to several neighbouring nodes, the best neighbor is characterized by the highest frequency value. The two loops stop when no more exploration can be done. To find all possible paths, we apply the following algorithm, called the *stringPath*() algorithm.

1. **allPaths()**

2. **SetList***C*; //components list

3. **SetList***P*; //paths list

4. **Integer***i*;

5. //Step 1

6. C:=allComponents(G);

7. //Step 2

8. **for***i***:= 1 to |***C***| do**

9. **begin**

10. $P_{i}:=stringPath(C_{i})\text{;}$

11. **end**

12. **return***P*;

By going through all connected components (determined by the *allComponents*() procedure), and due to the previous algorithm, a path *P*_*i*_ will be constructed for each connected component *C*_*i.*_

### Availability and requirements

Arapan-S is open access and freely available. All questions, comments and requests should be sent by email to nihon.sahli@gmail.com.

Project name: Arapan project

Project home page: http://shibuyalab.hgc.jp/Arapan/

Operating system(s): Windows, Linux (Ubuntu)

Programming language: C/C++

Other requirements: None

License: None required

Any restrictions to use by non-academics: None required

## Competing interests

The authors declare that they have no competing interests.

## Authors’ contributions

MS and TS conceived the research and wrote the article. MS conducted the research and implemented Arapan-S in C++ programming language. All authors have read and approved the final manuscript.
